# Improving the accuracy and robustness of carotid-femoral pulse wave velocity measurement using a simplified tube-load model

**DOI:** 10.1038/s41598-022-09256-z

**Published:** 2022-03-25

**Authors:** Lisheng Xu, Shuran Zhou, Lu Wang, Yang Yao, Liling Hao, Lin Qi, Yudong Yao, Hongguang Han, Ramakrishna Mukkamala, Stephen E. Greenwald

**Affiliations:** 1 College of Medicine and Biological Information Engineering, Northeastern University, Shenyang, China; 2 Engineering Research Center of Medical Imaging and Intelligent Analysis, Ministry of Education, Shenyang, China; 3 Neusoft Research of Intelligent Healthcare Technology, Co. Ltd., Shenyang, China; 4 School of Computer Science and Engineering, Northeastern University, Shenyang, China; 5 School of Information Science and Technology, ShanghaiTech University, Shanghai, China; 6 General Hospital of Northern Theater Command, Shenyang, China; 7 Department of Bioengineering, Department of Anesthesiology and Perioperative Medicine, University of Pittsburgh, Pittsburgh, USA; 8 Blizard Institute, Barts and The London School of Medicine and Dentistry, Queen Mary University of London, London, UK

**Keywords:** Biomedical engineering, Information technology

## Abstract

Arterial stiffness, as measured by pulse wave velocity, for the early non-invasive screening of cardiovascular disease is becoming ever more widely used and is an independent prognostic indicator for a variety of pathologies including arteriosclerosis. Carotid-femoral pulse wave velocity (cfPWV) is regarded as the gold standard for aortic stiffness. Existing algorithms for cfPWV estimation have been shown to have good repeatability and accuracy, however, further assessment is needed, especially when signal quality is compromised. We propose a method for calculating cfPWV based on a simplified tube-load model, which allows for the propagation and reflection of the pulse wave. In-vivo cfPWV measurements from 57 subjects and numerical cfPWV data based on a one-dimensional model were used to assess the method and its performance was compared to three other existing approaches (waveform matching, intersecting tangent, and cross-correlation). The cfPWV calculated using the simplified tube-load model had better repeatability than the other methods (Intra-group Correlation Coefficient, ICC = 0.985). The model was also more accurate than other methods (deviation, 0.13 ms^−1^) and was more robust when dealing with noisy signals. We conclude that the determination of cfPWV based on the proposed model can accurately and robustly evaluate arterial stiffness.

## Introduction

Cardiovascular Disease (CVD) is the world’s leading cause of death^1^. It has been estimated that by 2030, its global cost will reach $1440 billion^2^. From a pathophysiological point of view, the stiffness of the large elastic arteries near the heart is important as it determines the buffering ability of the arterial system to match the low output impedance of the heart to the higher input impedance of the peripheral circulation^3^. It is technically challenging to measure aortic stiffness locally by non-invasive approaches, but it is much easier to detect the pulse waveforms at peripheral sites.

Many studies have confirmed that arterial stiffness can be estimated non-invasively by measuring the pulse wave velocity (PWV)^4–7^. In these studies, the time delay between two arterial sites can be detected by relatively simple methods such as tonometry (pressure sensors), Doppler ultrasound or photoplethysmography^8^. By detecting the pulse wave from two specified arterial sites, the target PWV can then be obtained by measuring the distance between two sites divided by the measured time delay. Thus, accurate measurements of pulse transit time and distance are required. The latter can be obtained by MRI^9^, although this is impracticable for routine clinical measurements. In practice, the propagation distance is estimated from the measured body surface distance. According to the 2012 expert consensus document on the measurement of carotid-femoral pulse wave velocity (cfPWV), the direct distance between the carotid and femoral artery measurement sites multiplied by a factor of 0.8 is suggested as the standard for daily practice^10^.

The main methods for calculating arterial PWV are summarized in Table 1. They can be divided into three categories: point-based, waveform-based and model-based methods. In point-based methods, a particular feature of the wave serves as a fiducial or time-marker point to calculate the pulse transit time (PTT) between the two waves. The point-based method is less strongly affected by downstream reflections since the fiducial point is usually at or near to the start of systole or the “wave-foot”^11^. Various methods have been used to identify the wave-foot. These include finding the diastolic minimum, the maximum of the first derivative of the signal and the intersecting tangent algorithm^12,13^. The diastolic minimum method detects the minimum of each pulse wave and has been applied in commercial systems such as CAVI-Vasera VS-1000 (Fukuda Denshi, Tokyo, Japan)^14^ and pOpmètre (Axelife SAS, Saint-Nicolas-de-Redon, France)^15^. The maxima of the first or second derivative have also been used since the time at which they occur is closely related to that of the wave-foot and the second derivative approach is used in the Complior device (Alam Medical, Vincennes, France)^16^ and the Vicorder device (Skidmore Medical, Bristol, UK)^17^. However, some reports have questioned the accuracy of the diastolic minimum and the maximum derivative methods since they are susceptible to errors in identifying the foot of noisy signals^18–20^. To overcome the above weaknesses, the intersecting tangent algorithm was proposed which combines the diastole minimum method and the maximum of the first derivative methods and defines the fiducial point as the intersection of the horizontal line passing through the minimum and the tangent to the pulse wave at the point of its maximum first derivative. The expert consensus document^10^ recommends the use of the intersecting tangent algorithm on the grounds that it is more repeatable. This algorithm is widely used in commercial equipment such as PulsePen (DiaTecne, Milan, Italy)^21^ and SphygmoCor (AtCor Meddical, Sydney, Australia)^22^. In 2021, Buraioli et al.^23^ developed a new noninvasive system (the Athos device) for clinical PWV assessment based on the intersecting tangent algorithm. However, Salvi et al.^24^ found that different devices all using the intersecting tangent algorithm produced various cfPWV values when measuring the same artery.

**Table 1:** Summary of some methods for calculating arterial PWV.

Method	Algorithm	Definition	Advantages	Disadvantages
Point-based methods	Diastolic minimum	The foot is the minimum of arterial pulse waveform	Easy and simple to determine^12^	Susceptible to the noise and therefore of questionable accuracy^12^
	Derivative maximum	Marker point is the maximum of the first or second derivative	Easy to calculate^12,13^	Underestimate the reference PWV^25^
	Intersecting tangent	Fiducial point is the intersection of the first derivative maximum and a horizontal line passing through the diastolic minimum	Less susceptible to noise^19,24,26,27^	Have moderate agreement compared with the reference PWV^25^
Waveform-based methods	Waveform matching	Time-shift is obtained by minimizing the sum of squares error between a defined region of proximal and distal sites	Estimates PWV with high precision and low variability^25,28^	Susceptible to errors when large differences occur between waveform shape at the two sites^29^
	Cross- correlation	PTT is calculated by seeking the maximum of cross-correlation function between signals at two sites	Consider various frequency components of the pulse wave^29^	Effect on PTT of waveform differences between the two measurement sites not well characterized^30^
Model-based methods	Tube-load model	PTT is a model parameter which is estimated from aortic and peripheral pulse waveforms	Effect of wave reflection can be eliminated computationally^31^	Difficult to fully personalize model parameters^32,33^

The waveform-based method is an alternative approach to the determination of PWV is based on matching specified segments of the waveforms from each arterial site. This avoids the problem of identifying a specific point such as the foot of the wave and was described by McDonald as early as 1968^26^. A similar approach was described by Khir and Parker^27^. Recently, a modification of this this principle (termed ‘template matching’ and derived from image processing) has been adopted to extract repeated patterns in noisy pulse waveforms obtained by a non-contact optical technique (laser Doppler velocimetry), yielding promising results when applied to the problem of assessing signal quality and detecting artefacts^34^. In 2013, Vardoulis et al.^25^ proposed the diastolic patching method in which a section of the wave from one measurement site, taken from a region on either side of the diastolic minimum is correlated with a time-shifted window of the same length from the other measurement site. In 2015, a similar algorithm based on a defined region of the waveform was proposed by Hu et al.^28^. In a study on 81 human subjects, they showed that their approach was more reliable than the intersecting tangent method. Although these algorithms have good repeatability and accuracy, they do not consider whether and to what extent, the results are influenced by noise (i.e., robustness). Another waveform-based method is the cross-correlation algorithm^29^, which is less sensitive to noise and frequently applied to calculate the time delay and the similarity of two waveforms. Some studies have shown that the cross-correlation algorithm yields highly repeatable PWV measurements. However, the effect of differences in the waveform shape between the two measurement sites on the accuracy of PTT calculation is unknown^30^.

The tube-load model, a model-based method, has been proposed to obtain the PTT from measurement of pulse waveforms according to pulse wave transmission theory^31,32,35–37^. Such models consist of multiple parallel tubes with loads, which can represent the wave propagation and reflection phenomena of the arterial tree^38,39^. Each tube simulates the wave travel path between the aorta and peripheral arteries, while the load is the arterial bed distal to the peripheral artery. The tube-load model is often characterized by PTT, characteristic impedance, peripheral resistance and peripheral compliance^38^. The model parameters can be calculated by imposing the aortic and peripheral pressure waveforms as input and output of the model. PTT obtained in this way has been used to estimate PWV between the thoracic aorta and peripheral arteries^40^. Also, the relationship between PTT and blood pressure has been exploited to estimate blood pressure from proximal and distal pulse waveforms^32^. However, few studies have applied tube-load models to calculate cfPWV.

The purpose of this study is to investigate a simplified tube-load model for estimating the carotid-femoral pulse transit time (cfPTT) and to improve the accuracy and robustness of cfPWV measurement. Also, by comparing the results with several existing algorithms reported in recent years, the performance of the model is assessed for accuracy, repeatability, and robustness against signal noise. The contributions of this article are as follows. Firstly, the simplified tube-load model of arterial wave transmission and reflection is applied to calculate cfPWV. Secondly, considering the different direction of propagation of the carotid and femoral pulse waves, a dual tube and the single tube model are compared. Finally, the performance of the simplified tube-load model is evaluated by comparing experimental data from volunteers and numerical data generated by a one-dimensional (1D) arterial tree model.

## Methods

### Study subjects

In this study, both experimental and numerical data were used for the evaluation of the proposed algorithm and comparison with others. The differences between the dual and single tube-load models and the accuracy analysis of the four methods were assessed using numerical data. The repeatability and robustness analyses of the four methods were carried out using experimental data.

#### Experimental data

57 subjects aged 30.8 ± 15.2 years (ranging in age from 21 to 78 years) were recruited. The experimental protocol was approved by the Ethics Committee of the Northeastern University (EC-2020B017). All authors confirm that the research was performed in accordance with relevant guidelines and regulations. All subjects gave their informed consent and their basic characteristics are summarized in Table 2. The subjects were asked to relax for 15 min in a quiet room after which their weight and height was measured. Systolic blood pressure (SBP) and diastolic blood pressure (DBP) were measured three times using a sphygmomanometer and the average of the three measurements was used. Tonometric sensors SphygmoCor system (Model SCOR-Px, Atcor Medical, Sydney, Australia) and ECG electrodes were applied after the rest period and the carotid, femoral pulse wave and two-lead ECG signals were recorded using the SphymoCor PWV module.

**Table 2:** Subject characteristics (n = 57) Mean values ± SD.

	Male (n = 41)	Female (n = 16)
Age (years)	31.3 ± 14.0	30.2 ± 16.8
cf-distance (cm)	64.4 ± 10.1	55.2 ± 11.2
Height (cm)	174.9 ± 5.7	164.6 ± 7.7
Weight (kg)	72.7 ± 11.0	52.2 ± 7.4
BMI (kg/m^2^)	23.7 ± 2.8	21.0 ± 2.4
SBP (mmHg)	128.2 ± 10.4	118.0 ± 10.1
DBP (mmHg)	74.8 ± 8.9	63.1 ± 7.8
HR (beats/min)	66.9 ± 10.0	69.7 ± 7.5

Only the experimental data meeting the inbuilt quality control criteria (average pulse height, pulse height variation, diastolic variation, shape variation and operator index) of the SphygmoCor device were used. The operator index determined by its related quality control indices (built into the SphygmoCor system) is an indicator of the overall quality of the obtained waveform. It is calculated by assigning a weighting to the quality control indices (average pulse height, pulse height variation, diastolic variation and shape variation) and then adding them to give a number, expressed as a percentage. The higher the percentage, the higher is the quality of the waveforms obtained. (More details can be found in the SphymoCor XCEL operators manual^41^). In this study, we adopted the SphygmoCor quality criteria, based on the operator index value as follows: > 80%, Acceptable; 75–79%, Borderline; < 74%, Unacceptable. Pulse wave signals at the carotid and femoral sites were acquired sequentially, with simultaneous ECG recordings^27,42,43^.

Firstly, we put the right arm (RA) electrode of the ECG under the right clavicle, the left arm (LA) electrode under the left clavicle and the left leg (LL) electrode on the lower left abdomen. Finally, the pulse sensor was positioned on the neck over the right carotid artery. Simultaneous ECG and tonometer recordings were made for 30 s. Next, the pulse sensor was placed on the skin over the right femoral artery near the inguinal ligament and the ECG and tonometer recordings were made in the same way, again for 30 s. The ECG signals were used as a time reference for both recordings. The two recordings were deemed acceptable if the difference between the average heart rates calculated from them did not exceed 1 bpm. Three sets of valid data were collected sequentially by repeating the above steps three times. Finally, the straight-line distance between the carotid and femoral sites was measured to the nearest 2 mm with a tape over the body surface and the measured distance was corrected by multiplying by a factor of 0.8^28^.

Additional signal processing involved the removal of baseline drift using the wavelet transform^44^ and up-sampling of the raw signals collected at 128 Hz to 1 kHz by linear interpolation. The R peaks of the ECG signals were used to synchronise the carotid and femoral pulse waves, one beat at a time and the individual time delays were then averaged for all pairs of peaks obtained during the 30 s recording period. Finally, the cfPWV was calculated by dividing the measured distance between the two sites by the average time delay.

#### Numerical data

Currently, it is difficult to obtain the reference PTT in vivo, therefore, in-vivo evaluation of different methods for PWV estimation is not feasible^25^. In many studies, in-vivo data has been used only to assess the reproducibility or variability of different methods for PTT estimation. However, in-vitro or in-silico arterial models can experimentally or numerically evaluate the accuracy of different methods for PTT estimation.

Assuming 1D models of the arterial tree can accurately simulate pulse wave propagation^40,45–47^, such simulated data can then be used to evaluate the efficacy of PTT algorithms operating on in-vivo data; and we have adopted this approach here. Pressure waveforms in the ascending aorta recorded in patients undergoing cardiac catheterization were used as the input to the 1D model^48^, more details (see Supplementary Information) of which can be found^49–51^. The model was used to generate a range of waveforms by changing its parameters (i.e., vessel radius and length) within the physiological range^52^. Given that the four algorithms in this study are all based on the morphology of the waveform, we used the parameters which have the greatest influence on the waveform shape.

**Figure 1:**
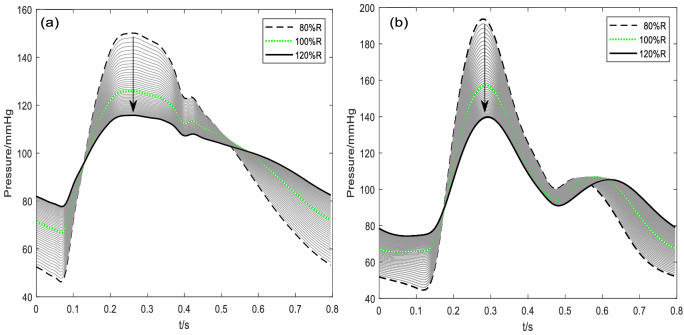
Effect of varying the model vessel radii on the morphology of the pulse waveform: (**a**) Right carotid artery, (**b**) right femoral artery. 80%*R*, 100%*R* and 120%*R* denote the minimum, starting and maximum radius, respectively. The arrows indicate the effect of increasing vessel radius. The x-axis is normalised time.

As an example, the radii of all arteries in the model were varied in small steps (1%) around a starting value within a range of ± 20%^25^, in which the starting value was the measured radius of the subject adopted from a previous study^49^. For example, starting with a femoral artery radius of 7.9 mm, it was then varied between 6.32 (7.9 × 80%) and 9.48 mm (7.9 × 120%), giving a total of 41 values. Figure 1a, b show the effect of changing the radii of the carotid and femoral arteries on their respective pulse waveforms.

### cfPTT calculation algorithms

#### Waveform matching

**Figure 2:**
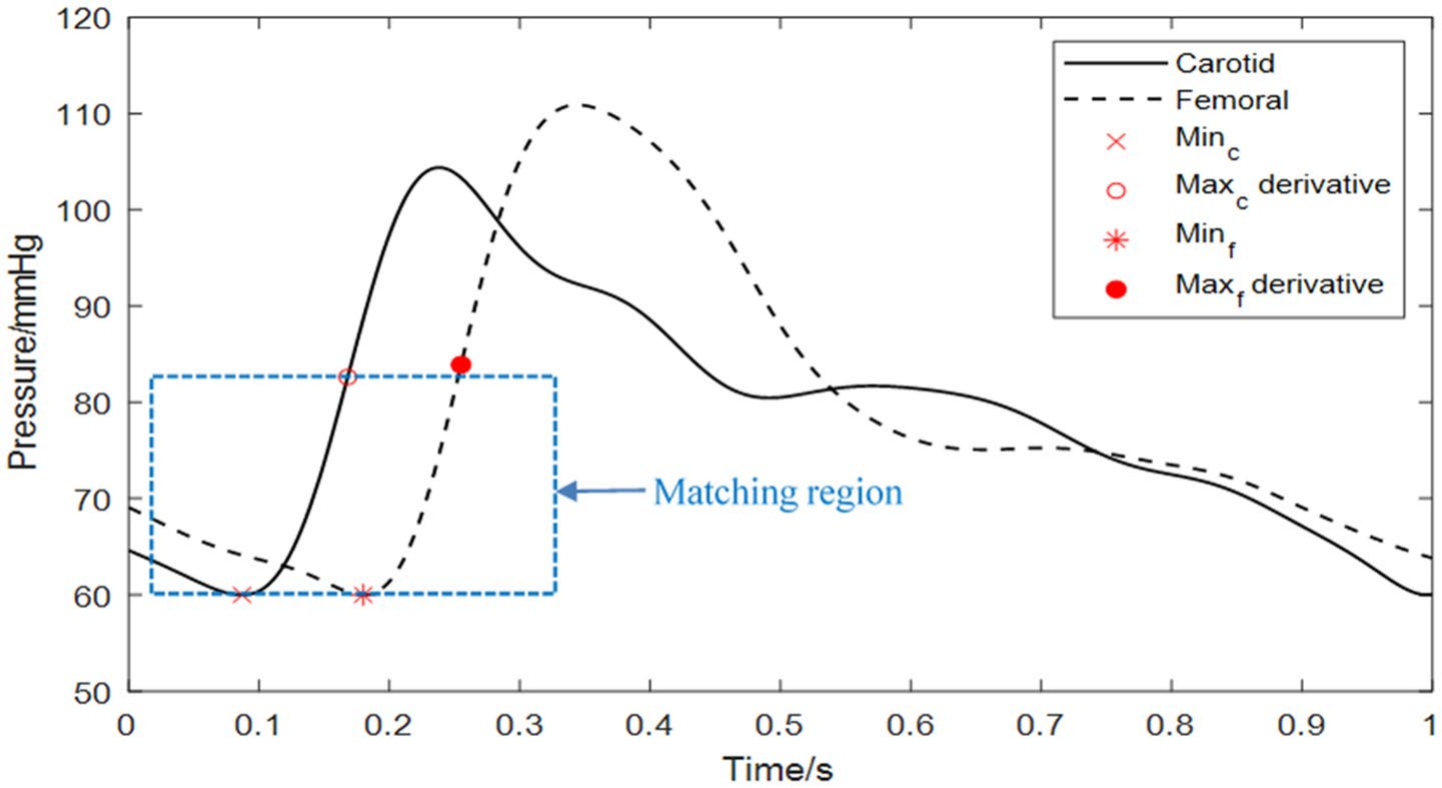
Graphical representation of the waveform matching method.

The waveform matching or diastole patch method is based on a characteristic region of the arterial pulse waveform instead of a characteristic point^25,28^. A segment of the waveform is chosen as the region over which the matching is performed. This region is defined for each beat, as being centred on the diastolic minimum and having a duration of 2*t*, where *t* is defined as the time between the diastolic minimum and the maximum of the first derivative during systole. The segment from the proximal (in this case, carotid) waveform is shifted in time in small steps and for each step is compared to a region of the same time duration in the distal waveform. The cfPTT between carotid and femoral waveforms is taken as the time shift which gives the minimum least squares error between the two waveforms, as shown in Fig. 2.

#### Intersecting tangent

**Figure 3:**
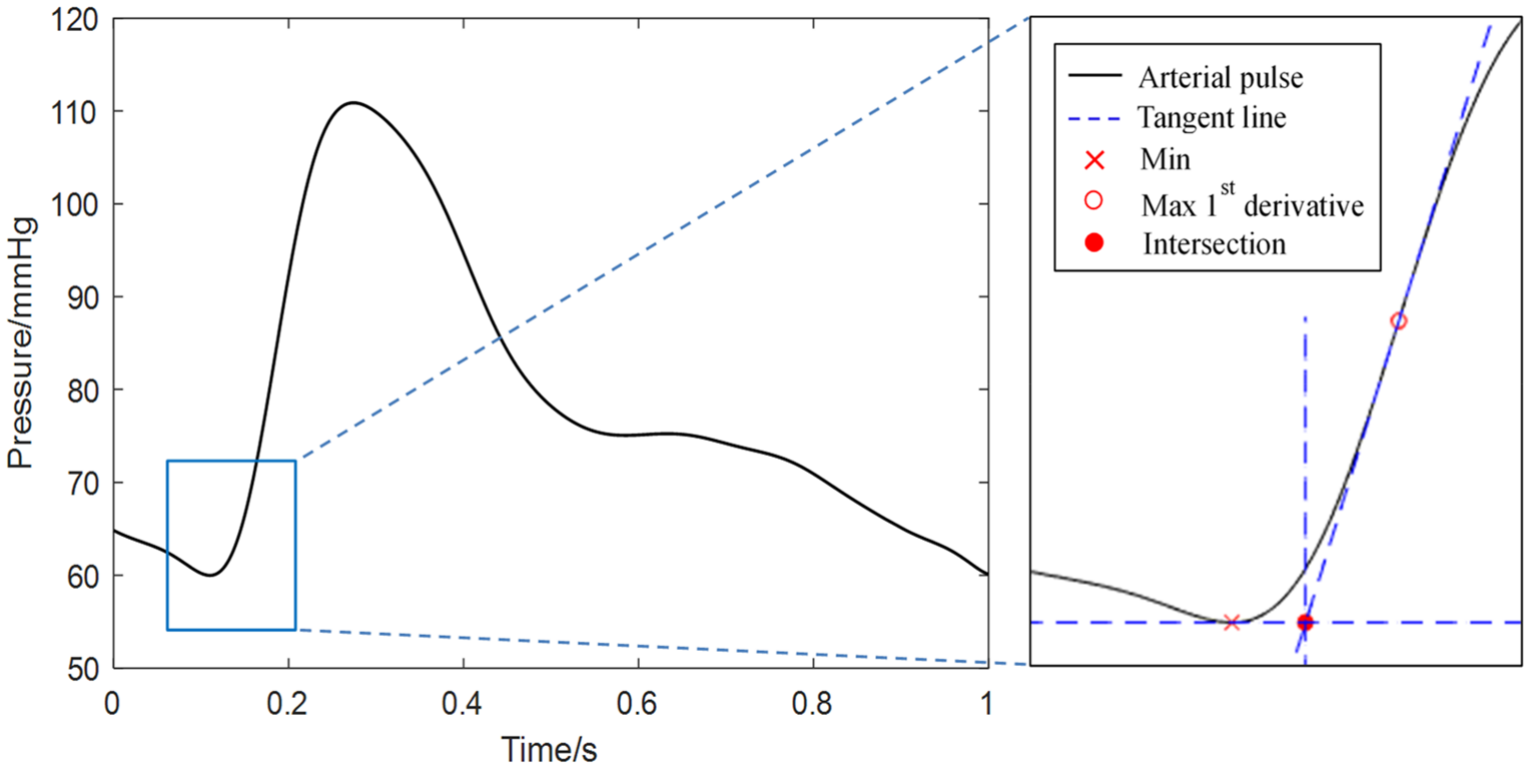
Graphical representation of the intersecting tangent method. The intersection point is taken as the fiducial point for determining PTT.

The intersecting tangent algorithm is one of the commonly used “foot-to-foot” methods^10,53^. The algorithm locates a characteristic point, defined as the intersection of two lines on the arterial pulse waveform. The first is the tangent to the maximum of the first derivative of the recorded signal during the systolic upstroke. The second is the tangent to the minimum of the recorded signal and is therefore a horizontal line parallel to the x-axis. Figure 3 shows how the process is used to identify the notional foot of the wave. The cfPTT between the carotid and femoral waveforms is taken as the time delay between their feet and is calculated for each heartbeat.

#### Cross-correlation

**Figure 4:**
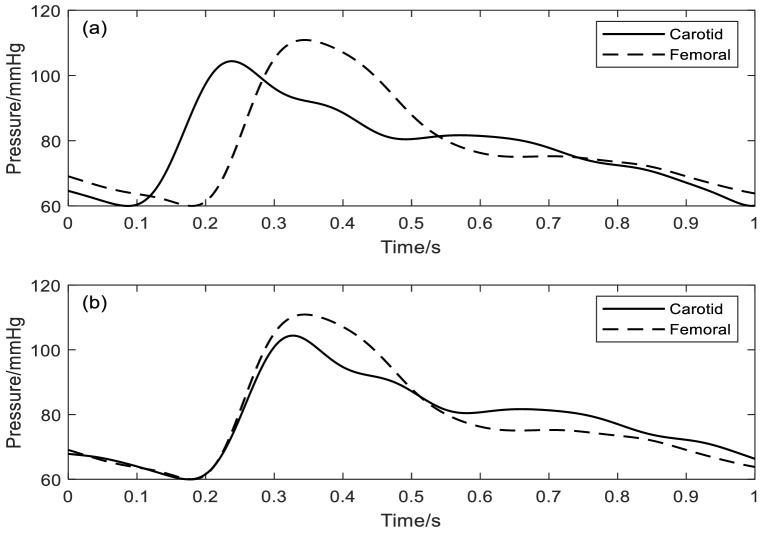
Graphical representation of the cross-correlation method showing its effect on a single heart beat: (**a**) the original carotid and femoral waveforms; (**b**) the femoral waveform having been shifted back in time by an amount determined by the maximum cross-correlation coefficient.

**Figure 5:**
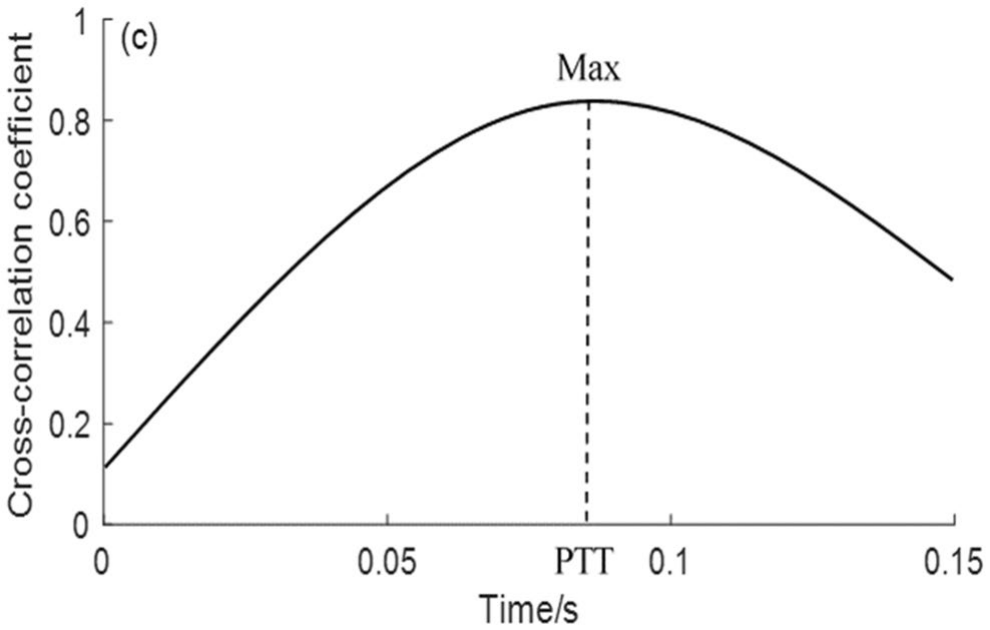
The cross-correlation coefficient against time shift.

Cross-correlation can be used to express the strength of association between two time series *x* (*t*) and *y* (*t*) at any two times *t*_*1*_ and *t*_2_^29^. In this case, to start with, the correlation coefficient is calculated between each pair of points from the carotid (*x*_*1*_, *x*_*2*_,…,*x*_*n*_) and from the femoral (*y*_*1*_, *y*_*2*_,…,*y*_*n*_) in a window encompassing, for instance the middle 80% of the recordings from the two sites. The calculation is repeated between *x*_*2*_ and *y*_*1*_, *x*_*3*_ and *y*_*2*_…*x*_*n*+*1*_ and *y*_*n*_, in effect having shifted the femoral signal back in time by one sample point. The process is repeated, generating a correlation coefficient for each time shift, until a maximum is found. The time shift at which this maximum occurs is taken as the PTT, as shown in Figs. 4, 5.

#### Tube-load model

**Figure 6:**
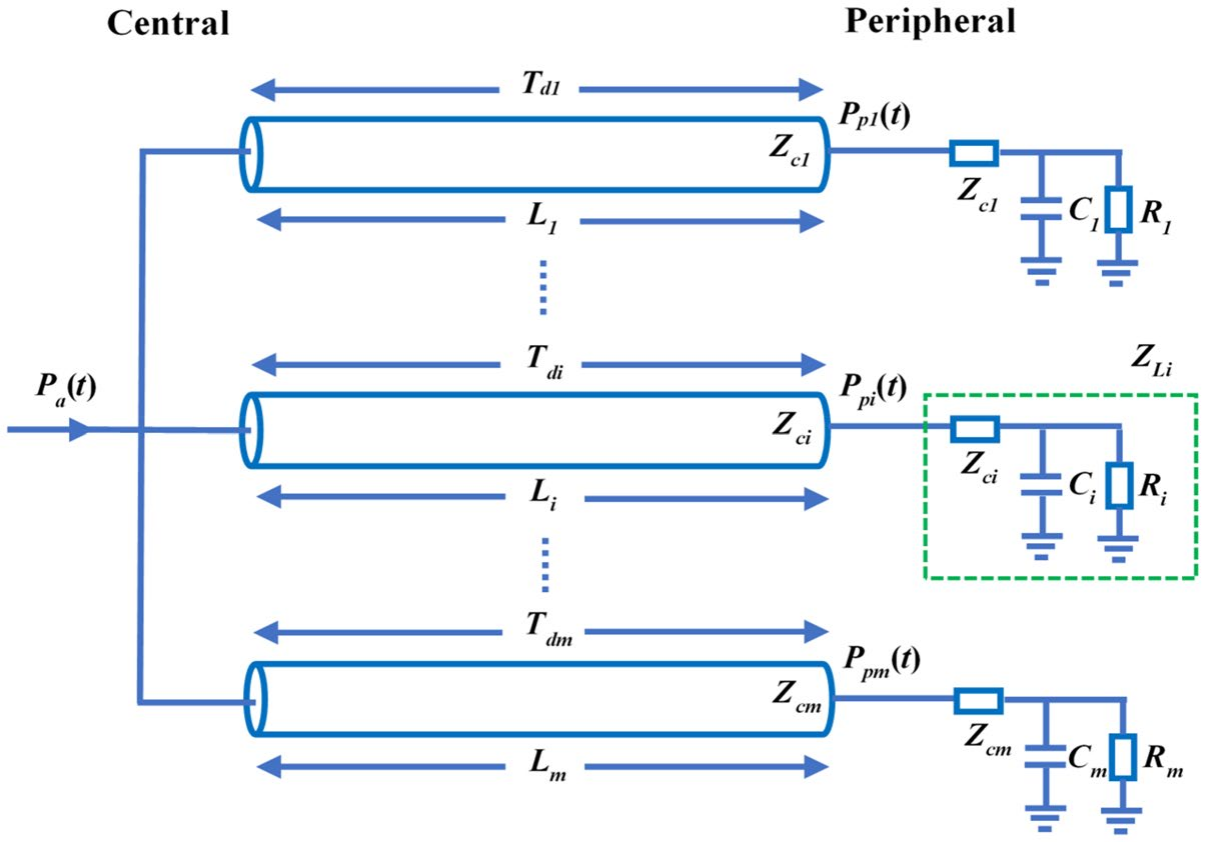
Arterial tube-load model for estimating PTT from the aorta to peripheral arteries. The *i* th tube (*i* = *1*,…, *m*) represents the wave travel path between the aorta and the *i* th peripheral artery. The terminal load *Z*_*Li*_ consists of characteristic impedance *Z*_*ci*_, resistance *R*_*i*_ and compliance *C*_*i*_. The PTT and the travel distance between the aortic *P*_*a*_(*t*) and peripheral *P*_*pi*_(*t*) pressure waveforms are defined as the time delay *T*_*di*_ and the vessel length *L*_*i*_, respectively. *T*_*dm*_, *L*_*m*_ and *P*_*pm*_(*t*) represent the time delay, vessel length and peripheral pressure in the *m* th tube, respectively. *Z*_*cm*_, *C*_*m*_ and *R*_*m*_ represent the characteristic impedance, compliance and resistance in the *m*th terminal load, respectively.

The tube-load model is composed of two main parts: tube and load^54^. It is assumed that the artery between the two measurement locations is a straight uniform tube, vessel curvature being ignored. It is also assumed that there is no energy dissipation along its length and therefore that there is no decay of the pulse wave within the tube. The system load mimics the load of all the small branches and micro-vessels^47,55^. The arterial system is described as a parallel connection of *m* such uniform and lossless tubes with terminal loads^42,56^, as shown in Fig. 6. The wave travel path between the aorta and the *i* th peripheral artery is referred to as the *i* th tube (*i* = *1*,…, *m*). Since the tube is lossless, each tube has constant characteristic impedance, as defined in (Eq. 1). The wave propagation time in the *i* th tube, *T*_di_ from the aorta to the *i* th peripheral artery is given by (Eq. 2). The three-element Windkessel model, including characteristic impedance *Z*_*ci*_, resistance *R*_*i*_ and compliance *C*_*i*_, is used as the terminal load *Z*_*L*_ Each pressure wave is considered as the summation of a forward and backward travelling component, the latter of which is due to reflections at the terminal load with a wave reflection coefficient, Γ_*i*_, for each tube, as defined in (Eq. 3).

1 $$Z_{ci} = \sqrt {\frac{{\rho L_{i} }}{{A_{i} C_{i} }}}$$

2 $$T_{ci} = \sqrt {\frac{{\rho L_{i} C_{i} }}{{A_{i} }}}$$

3 $$\Gamma_{i} (w) = \frac{{Z_{Li} (w) - Z_{ci} }}{{Z_{Li} (w) + Z_{ci} }} = \frac{{R_{i} C_{i} }}{{R_{i} C_{i} + 2Z_{i} C_{i} + jw2R_{i} C_{i} Z_{ci} C_{i} }}$$

where *ρ*, *L*_*i*_, *A*_*i*_, and *C*_*i*_ represent the blood density, length, area, and compliance of the *i* th tube, respectively.

**Figure 7:**
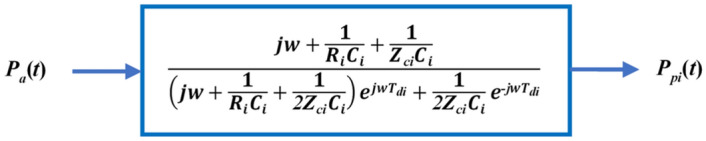
The transfer function between the aortic and peripheral pressure waveforms.

The transfer function between the aortic and peripheral arterial sites based on the tube-load model is established, as shown in Fig. 7. All model parameters (*T*_*di*_, *R*_*i*_*C*_*i*_, and *Z*_*ci*_*C*_*i*_) are estimated by fitting the aortic *P*_*a*_(*t*) and peripheral *P*_*pi*_(*t*) pressure waveforms as input and output of the tube-load model. A nonlinear least squares optimization method is applied to globally search for the optimal solution within the physiological range of each parameter. PTT from the aortic to peripheral arterial sites is obtained from the model parameter *T*_*di*_. PWV is calculated by dividing the distance *L*_*i*_ between proximal and distal arterial sites by PTT.

**Figure 8:**
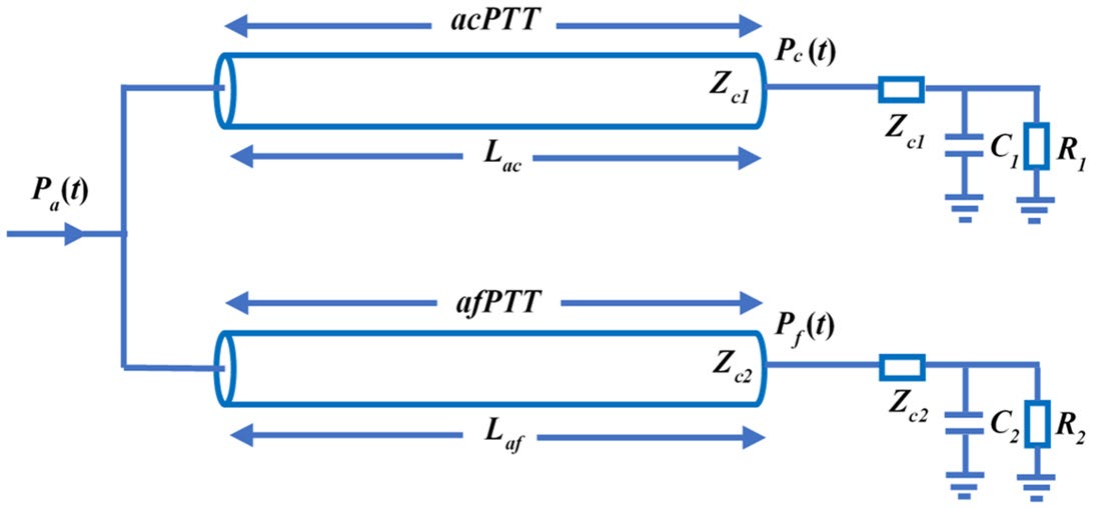
Parallel dual tube-load model for estimating PTT from the aorta to carotid and femoral arteries. The first tube represents the travel path between the aorta and carotid arteries. The second tube represents the travel path between the aorta and femoral arteries. The acPTT and vessel length *L*_*ac*_ represent the time delay and travel distance between the aortic *P*_*a*_(*t*) and carotid *P*_*c*_(*t*) pressure waveforms, respectively. The afPTT and vessel length *L*_*af*_ represent the time delay and travel distance between the aortic *P*_*a*_(*t*) and femoral *P*_*f*_(*t*) pressure waveforms, respectively.

Both the carotid and femoral arteries originate from the aorta; thus, it is reasonable to establish parallel dual tube models (ac-afTube). One tube represents the conduit from the aorta to the carotid artery (acTube) and the other, from the aorta to the femoral artery (afTube), as shown in Fig. 8. The pulse transit time from the aorta to the carotid arteries (acPTT) is calculated by fitting the aortic *P*_*a*_(*t*) and carotid *P*_*c*_(*t*) pressure waveforms. Similarly, the PTT from the aorta to the femoral arteries (afPTT) is calculated by fitting the aortic *P*_*a*_(*t*) and femoral *P*_*f*_(*t*) pressure waveforms. The difference between afPTT and acPTT (see Eq. 4) is the time delay between the carotid and femoral pulse waves, i.e., ac-afPTT. The ac-afPWV is calculated by dividing the distance between the carotid and femoral arterial sites with ac-afPTT.

4 $$ac - afPTT = \left| {afPTT - acPTT} \right|$$

**Figure 9:**
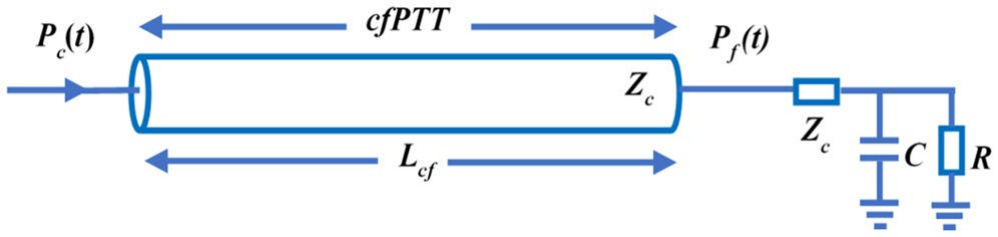
Simplified single tube-load model for estimating PTT from the carotid to femoral arteries. The tube represents the travel path between the carotid and femoral arteries. The cfPTT and vessel length *L*_*cf*_ represent the time delay and travel distance between the carotid *P*_*c*_(*t*) and femoral *P*_*f*_(*t*) pressure waveforms, respectively.

Directly establishing a single tube model between the carotid and femoral arteries (cfTube) is a simplification of the above parallel dual tube model, although it is obviously not truly representative of the arterial system. Nevertheless, this simplified single tube model may be workable because the cfPWV is often considered as a substitute for the aortic PWV, given that the carotid artery pressure is a reasonable approximation of the aortic pressure. In the simplified model, Fig. 9, a single tube represents the passage from a carotid to a femoral artery. The PTT between the carotid and femoral arteries (cfPTT) is then obtained by fitting the carotid *P*_*c*_(*t*) and femoral *P*_*f*_(*t*) pressure waveforms and the cfPWV is calculated by dividing the distance between the carotid and femoral arterial sites by cfPTT.

### Assessment of cfPWV calculation algorithms

The performance of the dual and single tube models was compared using the aortic, carotid and femoral pressure waveforms generated from the 1D model, which established that the two models gave similar results, the single tube model was then compared with the three existing methods described above, i.e., intersecting tangent, cross-correlation, and waveform matching. The evaluation analysis was carried out by comparing the repeatability, accuracy, and robustness of each method. The accuracy analysis was performed using carotid and femoral pressure waveforms from the 1D model while the in-vivo measured carotid and femoral pressure waveforms were used for the repeatability and robustness analysis.

#### Comparison of dual and single tube-load models

To determine if the cfPWV values obtained from the parallel dual tube-load model differed from those derived from the simplified single tube-load model, 1D simulation data were used. The aortic, carotid, and femoral pressure waveforms generated from the 1D model were used for the cfPWV calculation of the dual tube-load model. The dual tube-load model parameters (*Z*_*c1*_, *Z*_*c2*_, *C*_*1*_, *C*_*2*_, *R*_*1*_, *R*_*2*_, *acPTT*, *afPTT*) were estimated from the aortic, carotid, and femoral pressure waveforms generated from the 1D model using the system identification method. Similarly, the same carotid and femoral pressure waveforms of the 1D model were used for the cfPWV calculation of the single tube-load model. The single tube-load model parameters (*Z*_*c*_, *C*, *R*, *cfPTT*) were estimated from the carotid and femoral pressure waveforms using the system identification method. The agreement of the cfPWV values between the dual and single tube-load models was assessed by Bland–Altman analysis.

#### Repeatability analysis

Repeatability refers to the degree of similarity in the results obtained from successive measurements on the same subject under the same conditions. In this study, the intra-class correlation coefficient (ICC), a means of measuring and quantifying inter-observer and retest reliability^57^, was used to evaluate the repeatability of the four algorithms. The ICC is taken as the individual variability divided by the total variability to give a reliability coefficient. It is generally accepted that a value lower than 0.4 indicates poor reliability, and a value greater than 0.75 indicates good reliability^57^. In this study, the same operator collected three sets of data with duration of 30 s for each subject with a minimal delay between each run. Based on the three repeated measurements for each subject, the repeatability of the cfPWV calculated by the four algorithms was assessed. The in-vivo recordings of the carotid and femoral pressure pulse waves were used for the repeatability analysis.

#### Accuracy analysis

The accuracy analyses of the four algorithms were carried out using the carotid and femoral pressure pulse waves from the 1D model. A Bland–Altman plot was used to assess the agreement between the reference cfPWV value obtained from the 1D model and the cfPWV value calculated by the four algorithms. Additionally, the absolute differences between the reference cfPWV value and the cfPWV value of the four algorithms were expressed as mean ± SD.

#### Robustness analysis

Here, robustness, refers to the ability of the four algorithms to calculate cfPWV in the face of interference (in this case signal noise). Gaussian white noise was added to the in-vivo pulse wave signals^42^, to give signal-to-noise ratios (SNRs) of 20, 15, 10 and 5 (dB). The measured pulse wave signal was de-noised using the wavelet transform and this de-noised signal was considered as being effectively noiseless. The absolute errors between the noiseless and pulse wave signals with added noise were used to assess the robustness of each algorithm. The absolute errors of each SNR for the four algorithms were expressed in the form of bar-plots. Robustness analysis of the four algorithms was carried out using the carotid and femoral pressure pulse waves recorded in vivo.

### Statistical analysis

The results are expressed as mean ± SD. Bland–Altman plots were used to examine the agreement of the cfPWV values between the dual and single tube-load models. The agreement between the reference cfPWV by 1D model and estimated cfPWV by each algorithm was also assessed by Bland–Altman plots. Differences between the absolute errors of the various SNRs for each algorithm were assessed using the multi-sample non-parametric Friedman test. A linear regression analysis on the difference against the average cfPWV for the four algorithms was carried out. *P* < 0.05 was considered statistically significant. All statistical analysis was performed in SPSS Statistics 25 (IBM Corp., Armonk, NY, USA).

## Results

### Comparison of dual and single tube-load models

**Figure 10:**
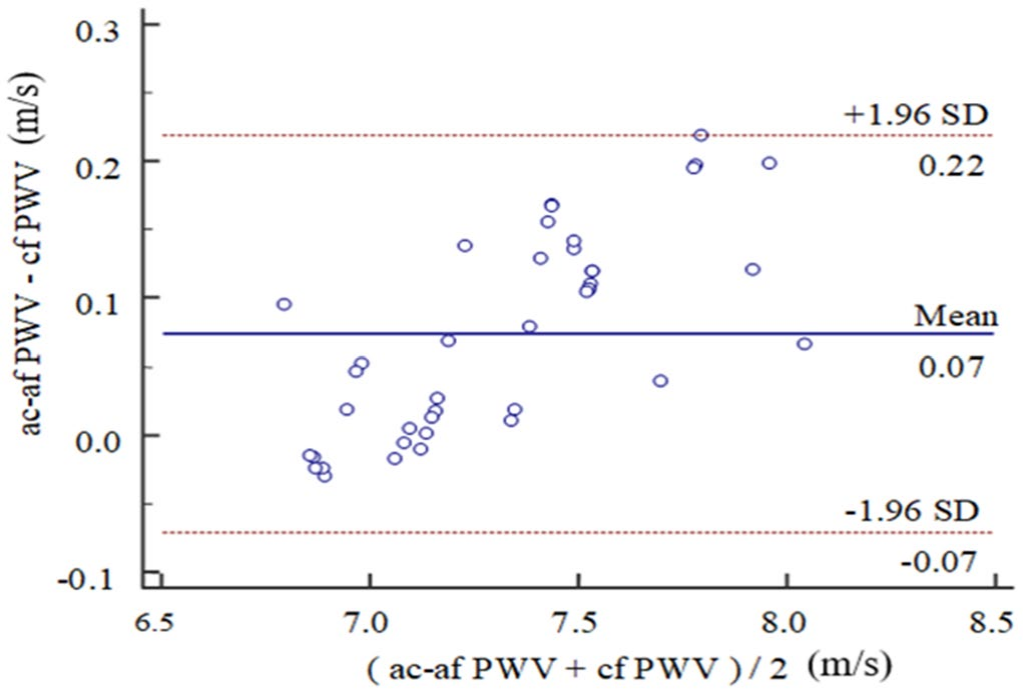
Bland–Altman comparison of ac-afPWV and cfPWV using the aortic, carotid and femoral pressure pulse waves from the 1D model.

The results of the ac-afPWV and cfPWV estimations from the parallel dual and simplified single tube-load models using the aortic, carotid and femoral pressure pulse waves from the 1D model are summarized in Table 3, and their comparison is shown as a Bland–Altman plot in Fig. 10. Table 3 shows that the ac-afPWV and cfPWV values from the two models are close (mean difference, 0.07 ms^−1^ and standard deviation, 0.05 ms^−1^). In Fig. 10, it is notable that most of the cfPWV values of the two models fall within the 95% confidence intervals and that the mean cfPWV difference is less than 0.1 ms^−1^, confirming that the two models are in good agreement.

**Table 3:** Comparison of the single and dual tube models using the 1D model data. The central aortic pressure waveform of a single subject served as the input for running the simulation of the 1D model.

Tube-load model	Mean (ms^−1^)	SD (ms^−1^)
cfPWV (single tube)	7.29	0.31
ac-afPWV (dual tubes)	7.36	0.36

The 41 groups of aortic, carotid and femoral pressure waveforms generated by changing the model parameters within the physiological range were used for calculating the means and SDs of PWV.

### Repeatability analysis

The results of the repeatability analysis for the four methods using the experimentally measured carotid and femoral pulse waveforms are summarized in Table 4. Although all four algorithms yielded highly repeatable results, the tube-load model gave the best performance (ICC = 0.99), followed by the waveform matching (ICC = 0.98), the cross-correlation (ICC = 0.95) and the intersecting tangent (ICC = 0.93) methods. Note that the mean values of cfPWV calculated by the four algorithms are different, ranging from 6.70 to 7.61 ms^−1^.

**Table 4:** The repeatability analysis for the four algorithms using the experimentally measured carotid and femoral pulse waveforms.

Algorithm	Mean (ms^−1^)	SD (ms^−1^)	ICC
Waveform matching	6.48	0.83	0.98
Intersecting tangent	6.70	0.74	0.93
Cross-correlation	7.61	1.51	0.95
Tube-load model	7.38	0.50	0.99

### Accuracy analysis

**Figure 11:**
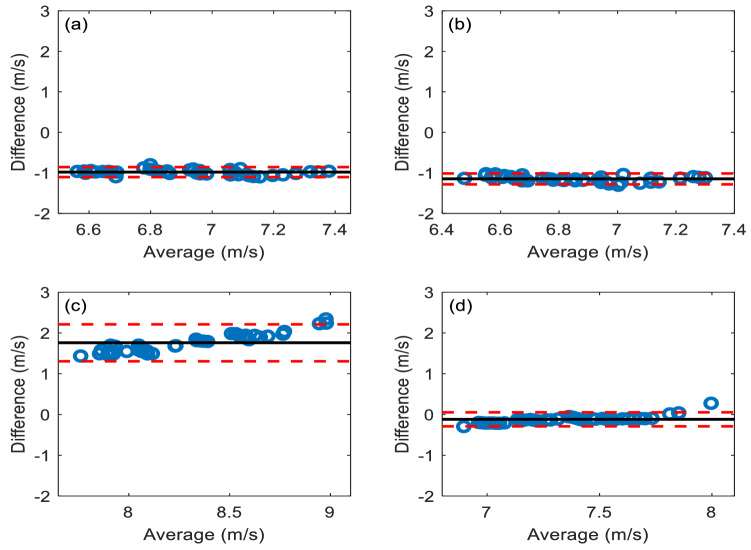
Bland–Altman comparisons of the reference cfPWV from the 1D model and the estimated cfPWV values of the four algorithms operating on the carotid and femoral pressure pulse waves from the 1D model: (**a**) Waveform matching; (**b**) Intersecting tangent; (**c**) Cross-correlation; (**d**) Tube-load model. The red dotted lines represent ± 1.96 standard deviation; the black solid line represents the mean difference.

Mean differences (± SDs) between the cfPWV values obtained by the 1D model and each of the four methods for deriving them using the carotid and femoral pressure pulse waves from the 1D model are shown in Table 5. In all cases, the deviation between the algorithms and the model was less than 1.8 ms^−1^, with the tube-load estimation giving much the closest value to the 1D model. From the Bland–Altman plots of Fig. 11, it is notable that for each algorithm, the majority of the cfPWV values fall within the 95% confidence intervals of the mean differences.

**Table 5:** Differences between the reference cfPWV given by the 1D model and estimated cfPWV of each algorithm using the carotid and femoral pressure pulse waves from the 1D model.

Algorithm	Mean (ms^−1^)	SD (ms^−1^)
Waveform matching	− 0.98	0.06
Intersecting tangent	− 1.15	0.07
Cross-correlation	1.76	0.23
Tube-load model	− 0.13	0.06

### Robustness analysis

**Figure 12:**
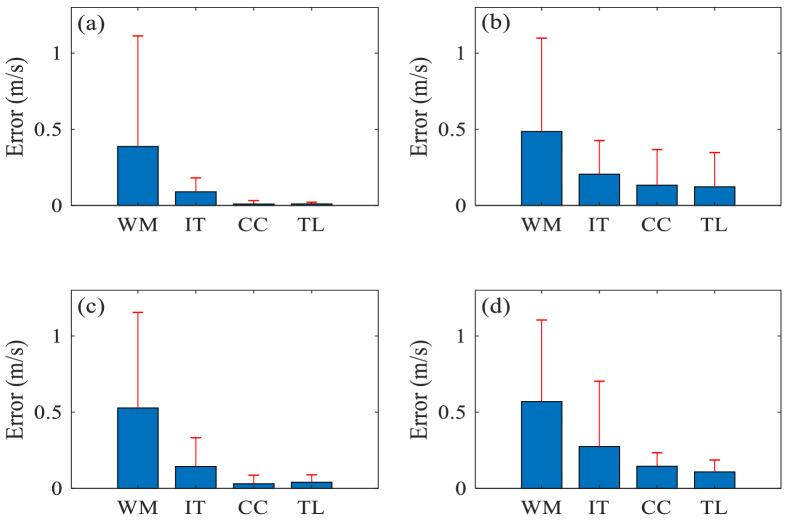
The effect of noise added to the experimentally measured carotid and femoral pulse waveforms on cfPWV calculated by each algorithm: (**a**) SNR = 20; (**b**) SNR = 15; (**c**) SNR = 10; (**d**) SNR = 5. WM, IT, CC and TL represent the waveform matching, intersecting tangent, cross-correlation and tube-load model methods, respectively.

Figure 12 shows the performance of the four algorithms on data containing different levels of noise using the experimentally measured carotid and femoral pulse waveforms. In general, the waveform matching method showed the poorest noise tolerance whatever the SNR, whereas the tube-load model and cross-correlation methods were more robust than the two other methods in the face of added noise. It is notable that tube-load model and cross-correlation methods are similarly robust.

Table 6 summarizes the mean difference (± SD) between cfPWV values of the noiseless signal and the signal with added noise over four SNRs for each method using the experimentally measured carotid and femoral pulse waveforms. The tube-load model method shows the smallest mean difference and SD. The waveform matching method has the biggest mean difference and the intersecting tangent method has the biggest SD. It is notable that the *P* values of the four algorithms (derived from the multi-sample non-parametric Friedman test) are all less than 0.001, which shows that there are significant differences between cfPWV values of the noiseless signal and the signal with added noise over the four SNRs for each algorithm.

**Table 6:** The mean difference between cfPWV values of the noiseless signal and the signal with added noise over the four levels of added noise for each algorithm using the experimentally measured carotid and femoral pulse waveforms.

Algorithm	Mean (ms^−1^)	SD (ms^−1^)	*P* value
Waveform matching	0.50	0.08	*P* < 0.001
Intersecting tangent	0.24	0.16	*P* < 0.001
Cross-correlation	0.08	0.07	*P* < 0.001
Tube-load model	0.07	0.05	*P* < 0.001

*P* < 0.001 indicates significant difference between cfPWV values of the noiseless signal and the signal with added noise over the different SNRs for each algorithm using the multi-sample non-parametric Friedman test.

The analysis of the repeatability, accuracy, and robustness of the four algorithms is summarized in Table 7. Repeatability and robustness analysis were performed using the experimentally measured carotid and femoral pulse waveforms. Accuracy analysis was carried out using the carotid and femoral pressure pulse waves from the 1D model. It is notable that the repeatability, accuracy, and robustness of cfPWV calculated by tube-load model algorithm are better than those derived from the other algorithms.

**Table 7:** Summary of the performance of four methods. Repeatability and robustness analyses were performed using the experimentally measured carotid and femoral pulse waveforms. Accuracy analysis was carried out using the carotid and femoral pressure pulse waves from the 1D model.

Algorithm	Repeatability	Accuracy	Robustness
	ICC	Mean (ms^−1^)	SD (ms^−1^)
Waveform matching	0.98	− 0.98	0.06
Intersecting tangent	0.93	− 1.15	0.07
Cross-correlation	0.95	1.76	0.23
Tube-load model	0.99	− 0.13	0.06

## Discussion

In this study, we have compared several current methods for determining cfPWV in terms of repeatability and robustness using the carotid and femoral pulse waves measured in vivo and, in terms of accuracy, using carotid and femoral pulse waves generated from a 1D model. It was found that these existing methods can estimate cfPWV values, with varying degrees of accuracy, to within 86.8% of the reference value. With the aim of improving upon these existing methods we have proposed a simplified single tube-load model. The results show that the proposed approach has good repeatability, accuracy and robustness when compared to the other methods of cfPWV calculation investigated here (see Table 7).

Four studies were carried out using both experimental and numerical data. The two sets of data do not conflict but are complementary. We used numerical data to evaluate the difference between the dual and single tube-load models and the accuracy of the four cfPWV calculation methods. Experimental data were used to evaluate the repeatability and robustness analyses of these methods. This is because the PTT value in vivo cannot yet be obtained accurately. As others have found, there are substantial differences in PWV values calculated by the different methods^8,25,46^. Although, in-vivo evaluation of the accuracy of cfPWV calculation is not feasible, a 1D arterial model of known vascular properties can be used to derive the true PWV analytically. Thus, in this study, numerical data were used to evaluate the difference between the two tube-load models and the accuracy of the four methods while at the same time the repeatability and robustness of these methods was evaluated.

The SphygmoCor system can be used to acquire signals simultaneously from two tonometric sensors. However, for this study, only one was available, so the pressure waveforms at each site were acquired sequentially. Although not ideal, this approach is commonly used^22,58,59^. To determine the cfPTT, two steps are required. The first is to simultaneously acquire the carotid pressure waveform and the ECG; and the second is the simultaneous acquisition of the femoral pressure waveform and ECG. In this way the ECG is used to synchronize the carotid and femoral pulse waves. A limitation of this approach is that the transit time may be influenced by heart rate differences between the two recordings resulting in errors. To minimize such errors, in this study the two recordings were considered acceptable only when the difference between the average heart rates calculated from them did not exceed 1 bpm. An ideal model of pulse wave propagation in the vascular system should include all vessels in the pathway under investigation. In this study, the aorta, carotid, and femoral arteries are involved so a dual parallel tube model, requiring the pressure waveforms from sites in these three vessels is a good starting point. However, since an accurate rendition of the aortic pressure waveform requires invasive measurements undergoing cardiac catheterization, a model based on a single tube (e.g., a notional carotid-femoral artery) needs only the carotid and femoral pressure waveforms, which can be obtained non-invasively, for instance by tonometry, and thus the aortic wave can be dispensed with. Here, using simulated input data, we have compared the outputs of the single and dual tube models and showed that they yield cfPWV values which agree closely (Table 3 and Fig. 10). We conclude therefore, that the simplified single tube model is an effective substitute for its more realistic dual tube counterpart.

In Fig. 10, there is a linear tendency for the difference between the ac-afPWV (parallel dual tube-load model) and cfPWV (simplified single tube-load model) estimations to increase as their average increases. A linear regression analysis gave the following relationship (*y* = − 1.09 + 0.16*x*, *P* < 0.05), showing that the difference between the ac-afPWV and cfPWV values increased significantly with the average of two values. Apart from speculating that the error arises because acPTT is calculated by fitting the aortic and carotid waveforms and afPTT by fitting the aortic and femoral waveforms, whereas the cfPTT is derived by fitting the carotid and femoral waves, (i.e. involving possible distance errors affecting the PWV values), we cannot offer a plausible explanation for this.

The cross-correlation (7.61 ms^−1^) and tube-load model (7.38 ms^−1^) methods gave higher estimates of PWV than the waveform matching (6.48 ms^−1^) and intersecting tangent (6.70 ms^−1^) methods as shown in Table 4. Vardoulis et al.^20^ reported similar findings. Table 4 also shows that the waveform matching and tube-load model had high repeatability, with ICC values of 0.98 and 0.99, respectively; whereas the intersecting tangent (ICC = 0.93) and cross-correlation (ICC = 0.94) methods had lower, although still good, repeatability. Whatever signal processing method is adopted, repeatability analysis, when used as a measure of reliability, implicitly assumes that the physiological state of the subject does not change between one set of measurements and the next. For this reason, it is generally found that measures of repeatability decrease with the time over which repeatability is assessed^60,61^. In this study, the repeatability was investigated over a period of minutes, thus the repeatability of all analysis methods was high. We note, though, that the tube-load model has the best performance. The marginally inferior performance of the intersecting tangent method may be ascribed to its reliance on a single fiducial point and that of the cross correlation, to the inherent problem of seeking a correlation between waveforms of different shape. It is notable that there is a linear tendency for the difference between the calculated methods and 1D model to increase with increasing cfPWV in Fig. 11c (cross correlation) and (d) (tube-load model), but not in Fig. 11a (waveform matching) and (b) (intersecting tangent). A regression analysis on the difference against the average cfPWV showed that the relationship was significant for the cross-correlation and tube-load model methods (cross-correlation: *y* = − 3.45 + 0.63*x*, *P* < 0.05; tube-load model: *y* = − 2.01 + 0.26*x*, *P* < 0.05), signifying that the difference between the reference and measured values increased with their average value. This may be due to reflections of the pulse wave. These reflection effects are expected to be smaller in the waveform matching and intersecting tangent methods because the position of the wave foot is determined primarily by the high frequency components of the wave and these high frequencies are less affected by reflections because they are more strongly attenuated by viscous damping than the lower frequencies^62^.

The waveform matching and intersecting tangent methods were more susceptible to noise than the cross correlation and tube-load model methods. Not surprisingly, these disparities suggest that, in addition to the timing of the pulses, the shape of the waveform will affect the measured PWV values. Similarly, the cfPWV calculation methods which rely on identifying a single point (intersecting tangent) and aligning sections of the pulse (waveform matching) rather than using entire pulses (cross correlation and tube-load model), are more sensitive to noise. Additionally, the tube-load model is based on a theory of the propagation and reflection of pulse waves. We can robustly obtain the cfPWV values even if the pulse waveforms are contaminated with noise (see Fig. 12 and Table 6).

The SD is larger than its mean in all 4 panels of Fig. 12, for the following possible reason. Gaussian white noise was added to the in-vivo pulse wave signals to assess the robustness of each algorithm. This follows a Gaussian distribution in amplitude and a uniform distribution in power spectral density^63,64^. Due to its random nature, the effect of the added noise on the cfPWV calculation is uncertain. In some subjects, this can lead to large absolute errors in cfPWV, which will result in comparatively large SDs. However, the overall mean is not greatly affected since there are only few large absolute errors and these large errors tend to be counterbalanced by comparable numbers of small values. Essentially, a large SD can result from the addition of a few outliers, whereas the overall mean would be largely unaffected.

## Limitations

There are several limitations to this study. The accuracy analyses of the four methods for calculating cfPWV and the difference between single and dual tube-load models were tested against the numerical data from a 1D model to avoid the practical difficulties of measuring reference cfPWV values. Inevitably, the numerical data used here will not fully reflect the properties of the real cardiovascular system, possibly leading to errors when evaluating all the algorithms. In all experiments, the carotid and femoral waveforms were measured sequentially with simultaneous ECG recordings for synchronization. Although the sequential measurements could lead to errors in the cfPTT estimation, analysis was performed only on signals in which the heart rate difference between the two sequential recording periods was less than 1 b.p.m. Nevertheless, when using the R peaks of ECG to align the carotid and femoral waveforms, there were some time differences between the two recordings due to short-term heart rate variability. In future work, we aim to record pulse signals from two sites simultaneously.

## Conclusion

In this study, we have applied a simplified single tube-load model to estimate cfPTT and therefore cfPWV. We found that the dual and single tube-load models gave consistent results, thus demonstrating that the single tube-load model can be used in place of the dual tube version with no loss of accuracy. Following this, the repeatability, accuracy, and robustness of four algorithms for calculating cfPWV were evaluated. For the cfPWV calculation, the simplified tube-load model had better repeatability (ICC: 0.985) than the other methods. The tube-load model method showed the highest accuracy when compared to the calculated reference value (deviation: 0.13 ms^−1^). For various signal-to-noise ratios, the tube-load model method was more robust than the existing methods. Therefore, it can be concluded that the overall performance of the tube-load model is superior to that of other methods assessed. The simplified single tube-load model can accurately and robustly provide a non-invasive approach to the measurement of cfPWV. In a future study we will investigate how well the method works when applied to signals obtained from a diverse range of subjects. The ideal way to evaluate any approach to the measurement of cfPWV is to test it against invasive measurements. In the absence of such a test, repeatability and resistance to noise are the most clinically useful way of judging the efficacy of any method.

## References

[1] World Health Organization. World health statistics 2021: Monitoring health for the SDGs, sustainable development goals. (2021).

[2] Roth GA (2017). Global, regional, and national burden of cardiovascular diseases for 10 causes, 1990 to 2015. J. Am. Coll. Cardiol..

[3] Greenwald SE (2002). Pulse pressure and arterial elasticity. Q. J. Med..

[4] Townsend RR (2015). Recommendations for improving and standardizing vascular research on arterial stiffness. Hypertension.

[5] Xiao H, Butlin M, Tan I, Avolio A (2017). Effects of cardiac timing and peripheral resistance on measurement of pulse wave velocity for assessment of arterial stiffness. Sci. Rep..

[6] Tavallali P, Razavi M, Pahlevan NM (2018). Artificial intelligence estimation of carotid-femoral pulse wave velocity using carotid waveform. Sci. Rep..

[7] Jiang Y (2007). The design and implementation of arteriosclerosis examination system based on PWV measurement. Autom. Instrum..

[8] Obeid H (2017). Numerical assessment and comparison of pulse wave velocity methods aiming at measuring aortic stiffness. Physiol. Meas..

[9] Houriez S (2019). Comparison of different methods for the estimation of aortic pulse wave velocity from 4D flow cardiovascular magnetic resonance. J. Cardiov. Magn. Reson..

[10] Van Bortel LM (2012). Expert consensus document on the measurement of aortic stiffness in daily practice using carotid-femoral pulse wave velocity. J. Hypertens..

[11] Nabeel PM, Kiran VR, Joseph J, Abhidev VV, Sivaprakasam M (2020). Local pulse wave velocity: Theory, methods, advancements, and clinical applications. IEEE Rev. Biomed. Eng..

[12] Vlachopoulos C, O’Rourke M, Nichols WW (2011). McDonald’s blood flow in arteries: Theoretical, experimental and clinical principles.

[13] Leloup AJA (2014). Applanation tonometry in mice: a novel noninvasive technique to assess pulse wave velocity and arterial stiffness. Hypertension.

[14] Kato A (2012). Brachial-ankle pulse wave velocity and the cardio-ankle vascular index as a predictor of cardiovascular outcomes in patients on regular hemodialysis. Ther. Apher. Dial..

[15] Salvi P (2019). Noninvasive estimation of aortic stiffness through different approaches: COMPARISON with intra-aortic recordings. Hypertension.

[16] Vivodtzev I (2013). Arterial stiffness by pulse wave velocity in COPD: Reliability and reproducibility. Eur. Respir. J..

[17] Fogacci F (2017). Effect of spontaneous changes in dietary components and lipoprotein (a) levels: Data from the Brisighella Heart Study. Atherosclerosis.

[18] Chiu YC, Arand PW, Shroff SG, Feldman T, Carroll JD (1991). Determination of pulse wave velocities with computerized algorithms. Am. Heart J..

[19] Millasseau SC, Stewart AD, Patel SJ, Redwood SR, Chowienczyk PJ (2005). Evaluation of carotid femoral pulse wave velocity. Hypertension.

[20] Hemon MC, Phillips JP (2016). Comparison of foot finding methods for deriving instantaneous pulse rates from photoplethysmographic signals. J. Clin. Monit. Comput..

[21] Salvi P (2004). Validation of a new non-invasive portable tonometer for determining arterial pressure wave and pulse wave velocity: The PulsePen device. J. Hypertens..

[22] Shahin Y, Barakat H, Barnes R, Chetter I (2013). The Vicorder device compared with SphygmoCor in the assessment of carotid-femoral pulse wave velocity in patients with peripheral arterial disease. Hypertens. Res..

[23] Buraioli I (2021). A new noninvasive system for clinical pulse wave velocity assessment: The Athos device. IEEE Trans. Biomed. Circuits Syst..

[24] Salvi P (2008). Comparative study of methodologies for pulse wave velocity estimation. J. Hum. Hypertens..

[25] Vardoulis O, Papaioannou TG, Stergiopulos N (2013). Validation of a novel and existing algorithms for the estimation of pulse transit time: Advancing the accuracy in pulse wave velocity measurement. Am. J. Physiol. Heart Circ. Physiol..

[26] McDonald DA (1968). Regional pulse-wave velocity in the arterial tree. J. Appl. Physiol..

[27] Khir AW, Zambanini A, Parker KH (2004). Local and regional wave speed in the aorta: Effects of arterial occlusion. Med. Eng. Phys..

[28] Hu FS (2015). A region-matching method for pulse transit time estimation: Potential for improving the accuracy in determining carotid femoral pulse wave velocity. J. Hum. Hypertens..

[29] Dauzat M (1996). Pulse wave velocity measurement by cross-correlation of Doppler velocity signals: Application to elderly volunteers during training. Int. J. Sports Med..

[30] Gaddum NR, Alastruey J, Beerbaum P, Chowienczyk P, Schaeffter T (2013). A technical assessment of pulse wave velocity algorithms applied to non-invasive arterial waveforms. Ann. Biomed. Eng..

[31] Gao M, Cheng HM, Sung SH (2017). Estimation of pulse transit time as a function of blood pressure using a nonlinear arterial tube-load model. IEEE Trans. Biomed. Eng..

[32] Mukkamala R (2015). Toward ubiquitous blood pressure monitoring via pulse transit time: Theory and practice. IEEE Trans. Biomed. Eng..

[33] Rashedi M (2013). Comparative study on tube-load modeling of arterial hemodynamics in humans. J. Biomech. Eng..

[34] Seoni S (2022). Template matching and matrix profile for signal quality assessment of carotid and femoral laser doppler vibrometer signals. Front. Physiol..

[35] Zhang G, Gao M, Mukkamala R (2011). Robust, beat-to-beat estimation of the true pulse transit time from central and peripheral blood pressure or flow waveforms using an arterial tube-load model. Conf. Proc. IEEE Eng. Med. Biol. Soc..

[36] Nejad SE, Carey JP, McMurtry MS, Hahn JO (2017). Model-based cardiovascular disease diagnosis: A preliminary in-silico study. Biomech. Model. Mechanobiol..

[37] Kim CS (2013). Quantification of wave reflection using peripheral blood pressure waveforms. IEEE J. Biomed. Health Inform..

[38] Rashedi M (2013). Comparative study on tube-load modeling of arterial hemodynamics in humans. J. Biomech. Eng..

[39] Swamy G, Xu D, Olivier NB, Mukkamala R (2009). An adaptive transfer function for deriving the aortic pressure waveform from a peripheral artery pressure waveform. Am. J. Physiol. Heart Circ. Physiol..

[40] Gao M, Zhang G, Olivier NB, Mukkamala R (2014). Improved pulse wave velocity estimation using an arterial tube-load model. IEEE Trans. Biomed. Eng..

[41] https://atcormedical.com/wp-content/uploads/simple-file-list/Downloads/SphygmoCor-XCEL-V1_3-Operators-Manual.pdf

[42] Mitchell GF (2010). Arterial stiffness and cardiovascular events: The Framingham heart study. Circulation.

[43] Peng RC, Li Y, Yan WR (2021). A correlation study of beat-to-beat RR intervals and pulse arrival time under natural state and cold stimulation. Sci. Rep..

[44] Xu L, Zhang D, Wang K (2005). Wavelet-based cascaded adaptive filter for removing baseline drift in pulse waveforms. IEEE Trans. Biomed. Eng..

[45] Qasem A, Avolio A (2008). Determination of aortic pulse wave velocity from waveform decomposition of the central aortic pressure pulse. Hypertension.

[46] Alastruey J (2011). Numerical assessment of time-domain methods for the estimation of local arterial pulse wave speed. J. Biomech..

[47] Alastruey J, Parker KH, Peiró J, Sherwin SJ (2009). Analysing the pattern of pulse waves in arterial networks: A time-domain study. J. Eng. Math..

[48] Yao Y (2017). Validation of an adaptive transfer function method to estimate the aortic pressure waveform. IEEE J. Biomed. Heal. Inform..

[49] Bessems D, Rutten M, van de Vosse F (2007). A wave propagation model of blood flow in large vessels using an approximate velocity profile function. J. Fluid Mech..

[50] Boileau E (2015). A benchmark study of numerical schemes for one-dimensional arterial blood flow modelling. Int. J. Numer. Methods Biomed. Eng..

[51] Kroon W, Huberts W, Bosboom M, van de Vosse F (2012). A numerical method of reduced complexity for simulating vascular hemodynamics using coupled 0D lumped and 1D wave propagation models. Comput. Math. Method M..

[52] Willemet M, Chowienczyk P, Alastruey J (2015). A database of virtual healthy subjects to assess the accuracy of foot-to-foot pulse wave velocities for estimation of aortic stiffness. Am. J. Physiol. Heart Circ. Physiol..

[53] Escobar-Restrepo B, Torres-Villa R, Kyriacou PA (2018). Evaluation of the linear relationship between pulse arrival time and blood pressure in ICU patients: Potential and limitations. Front. Physiol..

[54] Gao M (2016). A simple adaptive transfer function for deriving the central blood pressure waveform from a radial blood pressure waveform. Sci. Rep..

[55] Li X (2013). Why is ABI effective in detecting vascular stenosis? Investigation based on multibranch hemodynamic model. Sci. World J..

[56] Ghasemi Z (2018). Estimation of cardiovascular risk predictors from non-invasively measured diametric pulse volume waveforms via multiple measurement information fusion. Sci. Rep..

[57] Ionan AC, Polley MYC, Mcshane LM, Dobbin KK (2014). Comparison of confidence interval methods for an intra-class correlation coefficient (ICC). BMC Med. Res. Methodol..

[58] Butlin M, Qasem A (2016). Large artery stiffness assessment using SphygmoCor technology. Pulse.

[59] Butlin M (2013). Carotid-femoral pulse wave velocity assessment using novel cuff-based techniques: comparison with tonometric measurement. J. Hypertens..

[60] Loukogeorgakis S, Dawson R, Phillips N, Martyn CN, Greenwald SE (2002). Validation of a device to measure arterial pulse wave velocity by a photoplethysmographic method. Physiol. Meas..

[61] Wilkinson IB (2010). Artery Society guidelines for validation of non-invasive haemodynamic measurement devices: Part 1, arterial pulse wave velocity. Artery Res..

[62] Newman DL, Sipkema P, Greenwald SE, Westerhof N (1986). High frequency characteristics of the arterial system. J. Biomech..

[63] Van Den Broeck C (1983). On the relation between white shot noise, Gaussian white noise, and the dichotomic Markov process. J. Stat. Phys..

[64] Kafadar K (1986). Gaussian white-noise generation for digital signal synthesis. IEEE Trans. Instrum. Meas..

